# Pathways to effective surgical coverage in a lower-middle-income country: A multiple methods study of the family physician-led generalist surgical team in rural Nepal

**DOI:** 10.1371/journal.pgph.0001510

**Published:** 2023-02-28

**Authors:** Ollie Ross, Rabina Shakya, Rishav Shrestha, Shristi Shah, Amita Pradhan, Rahul Shrestha, Pushkar Bhandari, Becky Paris, Kashim Shah, Anil Shrestha, Mark Zimmerman, Hannah Henrikson, Suresh Tamang, Ruma Rajbhandari

**Affiliations:** 1 Nick Simons Institute, Lalitpur, Nepal; 2 University Hospital Southampton, Southampton, United Kingdom; 3 E3A Healthcare Pte.Ltd, Curie, Singapore, Singapore; 4 Galangoor Duwalami Primary Health Care Centre, Maryborough, Australia; 5 KIST Medical College, Lalitpur, Nepal; 6 BP Koirala Institute of Health Science, Dharan, Nepal; 7 All Nepal Hospital Pvt Limited, Samakhushi, Nepal; 8 Hereford County Hospital, Hereford, United Kingdom; 9 Patan Academy of Health Sciences, Lalitpur, Nepal; 10 Division of Global Health Equity, Brigham and Women’s Hospital, Boston, Massachusetts, United States of America; Universidad Nacional de Colombia, COLOMBIA

## Abstract

The Lancet Commission on Global Surgery (LCoGS) recommends using specialist surgical workforce density as one of 6 core indicators for monitoring universal access to safe, affordable surgical and anaesthesia care. Using Nepal as a case study, we explored the capacity of a generalist workforce (led by a family physician or MD general practitioner and non-physician anaesthetist) to enable effective surgical delivery through task-shifting. Using a multiple-methods approach, we retrospectively mapped essential surgical care and the enabling environment for surgery in 39 hospitals in 25 remote districts in Nepal and compared it with LCoGS indicators. All 25 districts performed surgery, 21 performed Caesarean section (CS), and 5 met at least 50% of district CS needs. Generalist surgical teams performed CS, the essential major operation at the district level, and very few laparotomies, but no operative orthopaedics. The density of specialist Surgeon/Anaesthesiologist/Obstetrician (SAO) was 0·4/100,000; that of Generalist teams (gSAO) led by a family physician (MD General Practitioners-MDGP) supported by non-physician anaesthetists was eight times higher at 3·1/100,000. gSAO presence was positively associated with a two-fold increase in CS availability. All surgical rates were well below LCoGS targets. 46% of hospitals had adequate enabling environments for surgery, 28% had functioning anaesthesia machines, and 75% had blood transfusion services. Despite very low SAO density, and often inadequate enabling environment, surgery can be done in remote districts. gSAO teams led by family physicians are providing essential surgery, with CS the commonest major operation. gSAO density is eight times higher than specialists and they can undertake more complex operations than just CS alone. These family physician-led functional teams are providing a pathway to effective surgical coverage in remote Nepal.

## Introduction

Universal coverage of essential surgery is a key component of universal health coverage and requires service provision, quality, and access [1, 2]. Low- and middle-income countries (LMICs) in particular face many challenges as they implement universal coverage of essential surgery, particularly for their disadvantaged populations [3–10].

The 2015 Lancet Commission on Global Surgery (LCoGS) and Disease Control Priorities: Essential Surgery (DCP3) have provided indicators, targets, and pathways to guide analysis and planning for national surgical services [11–13]. For example, they have proposed 6 core indicators for monitoring universal access to safe, affordable surgical and anaesthesia care with “specialist surgical workforce density” being an important one. This is the number of working specialist surgical, anaesthetic, and obstetric physicians (SAO) per 100 000 population. Demand and supply studies are appearing, including in Nepal, but few studies measure and outline successful models of district surgical delivery [14].

Nepal’s remote districts are some of the hardest to reach and most medically underserved in the world. Mountainous terrain, poverty, access restrictions, and poor retention of key health workers continue to hinder the Government of Nepal’s provision of essential surgical services in rural areas. Effective coverage is the proportion of people in need of services who receive services of sufficient quality to obtain potential health gains and is a core, though very difficult to measure, concept of universal surgical coverage [13]. Provision of effective surgical coverage to these populations remains the highest challenge nationally. Nepal’s current state of surgical delivery in remote settings is unclear and further analysis of the state of surgery is hampered by inconsistent metrics and local documentation. There has been very limited ‘on the ground’ research and currently, there is no comprehensive surgical data tool in use in Nepal.

One thing that does seem to set Nepal apart from other LMICs is the generalist surgical teams working in rural areas. These teams are composed of the MDGP (Medical Doctorate in General Practice), Advanced Skilled Birth Attendant trained Medical Officers (ASBA-MO), and non-doctor anaesthesia providers—Anaesthesia Assistants [15]. We refer to health workers in these generalist surgical teams as “gSAOs” in this paper, mirroring the Lancet definition of SAOs.

Nepal’s MDGP program was established as a postgraduate program specifically seeking to address the rural doctor shortage by training doctors for work in government district hospitals [15]. Health policymakers realised that Nepal needed “generalist” physicians who could cope with the wide range of preventative and curative medicine required in rural areas. Compared to general practice (or family medicine) doctors in the US or UK, Nepal’s GP doctors are post-graduate doctors trained at medical universities in Nepal to provide not only primary care but crucially, emergency life saving surgical and obstetric operations such as caesarean sections, appendectomy, orthopaedics, and laparotomy [15]. The MDGP program has been in place with training, posting, and service in rural areas supported by the Government of Nepal for nearly 40 years [15]. A study conducted in 2006 followed all MDGPs trained in Nepal between 1982 and 2005 (n = 99) and found 87 MDGPs were still in Nepal, of whom 61% were working outside of the capital city of Kathmandu and 35% were working in government facilities [16].

In addition to the MDGP, medical officers with an MBBS degree may be trained to carry out CS in the advanced skilled birth attendant course (ASBA-MO). The ASBA training is an in-service training carried out by the National Health Training Centre of Nepal; as of 2021 around 234 Medical Officers have received ASBA training. For CS, the MDGPs may be assisted or deputised by Medical Officers (MO) with advanced SBA level (ASBA) training; the MO and MDGP doctors may even be the only surgical provider and at times, may even be the sole anaesthesia provider [17–19].

In the relative absence of medical anaesthesiologists at the rural level, Nepal has turned to non-doctor anaesthesia providers to meet the gap: so-called Anaesthesia Assistants (AAs) [20]. GoN nurses and other paramedics have been trained on a 6-month AA training course (2002–2011) or since 2011, a 12-month upgraded AA course with entry limited to higher-trained cadres—staff nurses and health assistants. In 2014, an annual survey of 100 AAs found that 81% of these AAs were still working in hospitals capable of providing surgical services [20].

In this study, we explored surgical volumes, human resources for surgery (specialist vs. generalist), the enabling environment for surgery, and barriers and solutions to increasing surgical access in 25 of the most remote and rural districts of Nepal. We compared these district-level metrics to global standards to build a map of successful pathways to effective surgical coverage in Nepal, particularly exploring the role of generalist surgical teams (gSAO). To our knowledge, this is the first systematic, comprehensive (covering 33% of the districts in Nepal and 3.6 million people), site-based “on-the-ground” study to map current surgical activity in rural Nepal. While our study only focuses on Nepal, we hope that the results generated from this study will help inform successful pathways to surgical coverage in low- and middle-income settings globally.

## Materials and methods

### Ethics statement and data availability statement

Ethical approval was obtained from the Nepal Health Research Council (No. 90/2016) for the data collection and questionnaire design. The investigators sought verbal informed consent before enrolling study participants. The investigators provided information about the study’s objective to the study participants (key informants) using the local language. The participants were assured complete confidentiality. The datasets generated during and/or analyzed during the current study are available from the corresponding author on reasonable request.

### Methods

**Fig 1:**
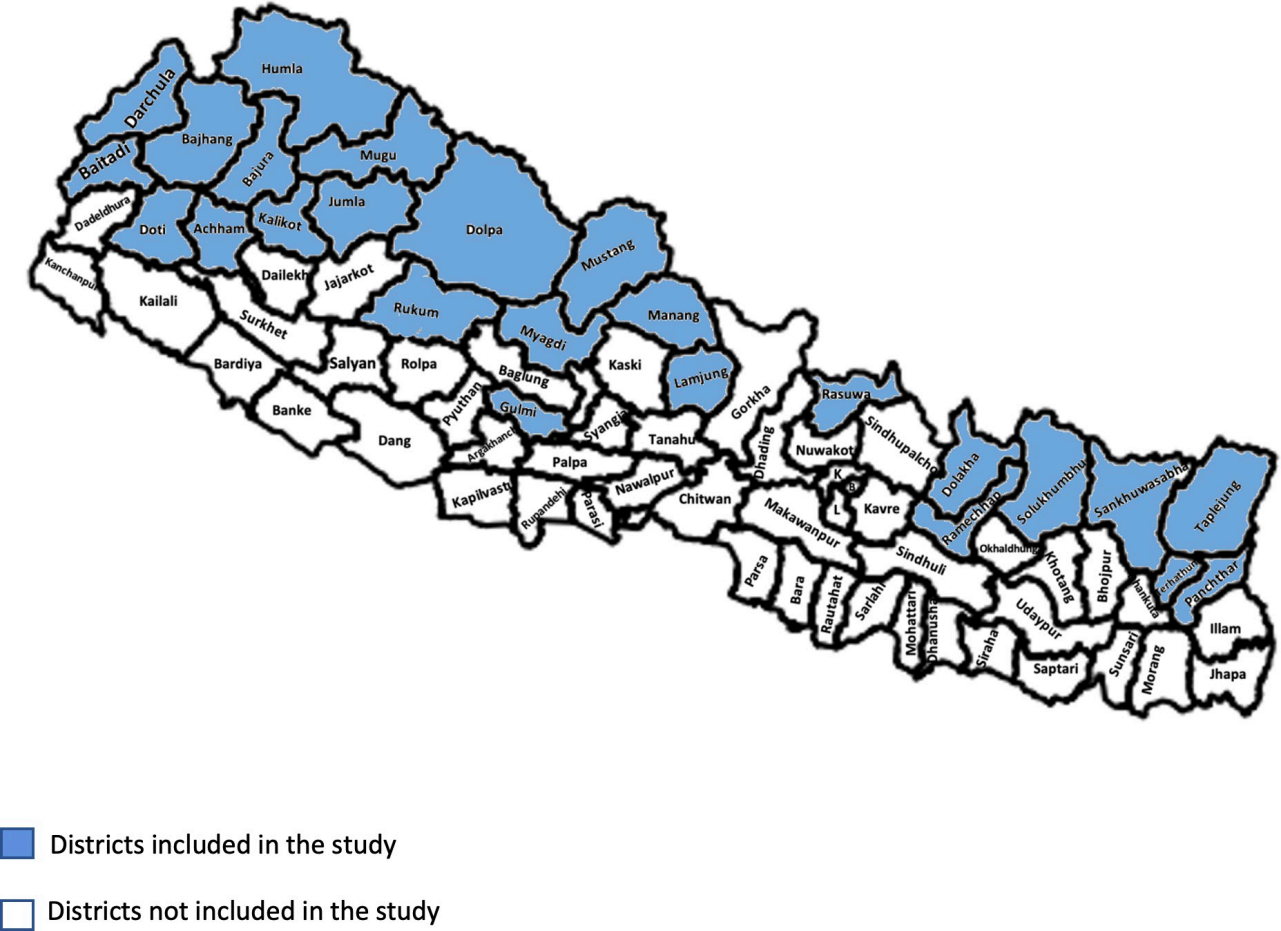
Map of districts selected for the study (blue). (A) License: Creative Commons Attribution–ShareAlike 4.0 International License. Caption Credit: geoBoundaries-NPL-ADM2-PREVIEW.png (640×480) (raw.githubusercontent.com). (Accessed December 28, 2022), and Runfola, D. et al. (2020) geoBoundaries: A global database of political administrative boundaries. PLoS ONE 15(4): e0231866. https://doi.org/10.1371/journal.pone.0231866. (Accessed December 28, 2022) [21, 22].

Of Nepal’s 75 districts, 25 remote and rural districts were chosen- these were defined as those with low expected surgical activity, where the entire district population lives more than two hours from a surgical hospital in another district (to match the LCoGS indicator of 2-hour access) and into which patients seldom come from other districts for surgery, thus ensuring stable population denominators. All 39 hospitals in those districts listed by DOHS were selected for field visits [Fig 1].

A multiple methods approach was employed using a study data tool developed from validated national [23, 24], and international sources [2, 12, 25], and refined after piloting. The data tool was designed specifically for Nepali hospitals by integrating recognised international standards with Government of Nepal standards and assessment tools. International sources were the Lancet Commission on Global Surgery, Disease Control Priorities 3 and WHO Situational Analysis Tool and adapted to Nepal’s hospitals using the Government of Nepal Minimum Services Standards and Government of Nepal National Health Training Centre Follow Up and Enhancement tools. Field visits were undertaken by two trained medical officer researchers visiting each hospital between July-October 2016. The operation theatre (OT) logbook was used as the absolute data source for operations performed. All operations deemed indicative of a functional surgical service at district level [2] were recorded for the fiscal year July 2015-July 2016 and included the three LCoGS Bellwether procedures: (CS, laparotomy, and treatment of open long bone fracture) [26, 27]. All operations were subsequently categorised into major and minor operations according to GoN definitions [see S1 Table].

Surgical readiness (enabling environment) was assessed by recording the human resources for surgery: both specialist (SAO) and generalist SAO (gSAO) teams. Each surgical facility was assessed against a detailed checklist for surgery including OT equipment, anaesthesia equipment, drugs, surgical instruments, and support services. The checklist was built from Nepali and international surgical assessment tools. Items on the checklist were recorded by the researchers as present and functional, or absent according to a report by the OT nurse or doctor. Access to essential operations was calculated as the percentage of the overall total study population with the Bellwether procedure having been performed in their district. Hospital surgical mortality and referral data were recorded if available. Additional calculated indicators were district met need for CS (based on a predicted WHO minimum rate of 5% of live births) [11, 28].

We aimed to further elucidate and verify the quantitative findings from the survey through qualitative questions asked in a short interview format at the end of the survey to key respondents. The key respondents were purposely selected–MO, MDGP and Medical Superintendents–who were lead surgical staff for the selected hospitals. Qualitative questions were designed to have better understanding of rural district surgical needs/expectations, gaps/barriers and solutions/recommendations from the perspective of lead clinicians. Three main questions were asked of the selected hospitals:

- What operations do you think should be done here?
- What do you think are the barriers to doing more surgery here?
- What are the solutions to doing more surgery here?

Data were analysed manually for emerging themes. Researcher field notes and memos were also reviewed. This triangulation complemented and supported the quantitative findings of the study.

Quantitative findings were compared to four LCoGS 2030 targets: preparedness (access to surgery, human resources for surgery), and delivery (surgical volume, perioperative mortality). All quantitative data were collected in Microsoft Excel and stored in SPSS 28.0.1 (IBM, Armonk, NY) prior to final formatting and analysis in Stata Version 16 (StataCorp, College Station, TX). Financial impacts (reduction in impoverishing and catastrophic expenditures) were not recorded in this study.

We used Fisher’s exact test to conduct between-group comparisons for categorical variables. We performed univariable and multivariable logistic regression to identify independent factors associated with a hospital’s performance of Caesarean sections.

## Results

### Hospital demographics

Districts represented 50% of Nepal’s geographical area, 33% of Nepal’s districts, and 14% of Nepal’s population with a total study population for all 25 districts of 3,605,796. Median district population was 141,652 (range 6399–269,573). 17(68%) districts were below the national mean HDI score of 0·49.

Of all 39 hospitals visited, two-thirds (n = 26) of the hospitals were district or district-level government hospitals. More than half of the hospitals (54%) were 15-bedded district hospitals, and an additional 13% were upgraded district-level or 50-bedded hospitals. Nearly three-quarters of these government hospitals received support for surgical services (primarily CS) from either the GoN’s CEONC program or other organisations.

The focus of our study was to address those populations most excluded from surgical access. In 2016, the districts of Nepal could be divided into those that either possess specialty (referral) surgical services or that have close (< 2-hour travel) access in the adjacent districts (n = 50) and those districts whose entire populations lie more than 2 hours from such surgical centres. The latter were our selected study districts (n = 25). We mapped times from each district’s main hub town to the closest known surgical facility outside that district. All districts were greater than two hours from a known surgical referral centre, the median being 7.3 hours [see S2 Table]. In addition, all districts studied were hilly and mountainous, and it is certain that patient travel times to the district hub or even beyond it would be prolonged and slow.

Detailed geospatial mapping of the population who live within 2h of Bellwether capable hospitals (those in our study or those beyond the district hub) would be very useful but could not be undertaken for this study [29, 30].

### Surgical volumes

**Fig 2:**
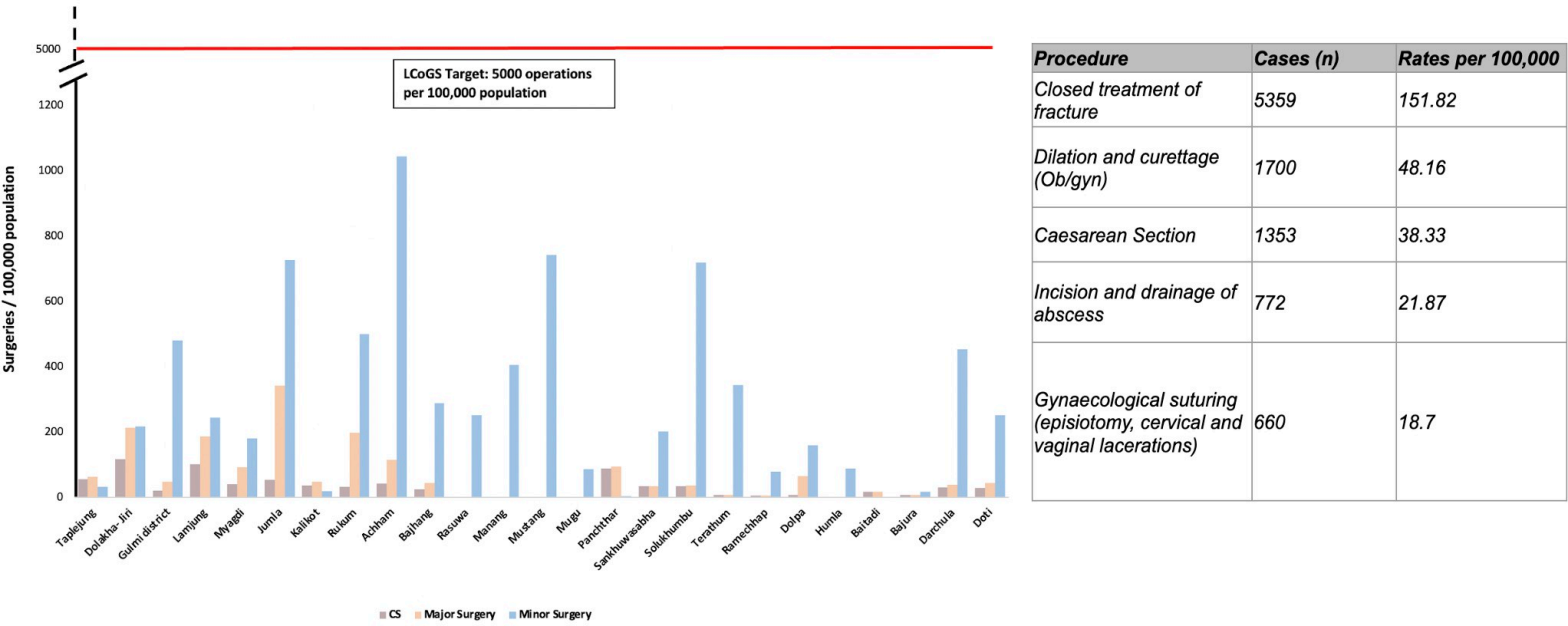
Surgical rates of bellwether procedures per study district, compared to lancet commission goals, and top 5 major operations performed in study district hospitals.

All 25 districts had a hospital performing some surgeries; of these, 21 districts had at least one hospital performing CS. Operative rates for all districts combined were 387 total operations/100,000 population and 80 major operations/100,000 population [Fig 2]; the LCoGS 2030 target indicator is 5000 total procedures or operations/100,000 population [1]. Most surgery was minor, and most hospitals performed very few operations [Fig 2, S1 and S2 Figs]. CS made up the majority of major operations performed; other common operations were closed treatment of fracture, dilation and curettage, abscess drainage, and gynecological suturing [Fig 2, S3 Fig].

#### Caesarean sections

Of the Bellwether procedures, CS was most commonly performed (n = 1353) accounting for 5% of all operations. CS was performed in 27 hospitals in 21 districts (84%). Some districts had multiple hospitals performing CS, whereas four districts had no CS performed at all. The median annual rate per hospital is 22 with a wide range and outliers. The met need for CS in each district was calculated as CS performed in that district from the study divided by the predicted WHO minimum CS rate of 5% of live births in that district. Five districts achieved at least 50% of met need; three of these exceeded 75% of met need.

#### Laparotomy

Laparotomy was performed in extremely low numbers (60 operations overall). 13 gastro-intestinal (GI) laparotomies were performed in total, and the remainder were gynaecological laparotomies. In five districts, one or more GI laparotomies were performed, but in the remaining 20 districts, no GI laparotomies were undertaken. However, in three of the five districts, GI laparotomy had been successfully performed by a gSAO team (an MDGP supported by a non-doctor anaesthesia provider (AA)) in a government district hospital. The GI laparotomy rate is 0·4/100,000 for all districts’ populations. As the surgical burden of disease requiring laparotomy is not known, met need cannot be calculated. However, the incidence of peritonitis and bowel obstruction has been estimated to be as high as 1,364 per 100,000 population which gives some light to the unmet need for laparotomy in this population [31].

#### Appendectomy and other major operations

Appendectomy, an emergency operation, was also performed but in low numbers (248 operations overall). The appendectomy rate was 7·03/100,000 for all districts’ populations, over ten times lower than rates typically observed in high-income countries (~80/100,000) [29]. However, 40.7% of these were performed by gSAO teams. These operations may be ‘stepping stones to higher capability (see section on Stepping Stone operations below).

Other major elective operations (e.g. hysterectomy) were performed only by visiting surgical teams (“camps”). Visiting teams were mostly orthopaedic or gynaecology in nature. Such teams were usually organised as short, one to two week-long camps doing primarily elective surgeries e.g. vaginal hysterectomies. From the operating theatre records, it was not possible to distinguish what was done by visiting surgeons and local teams but camps were infrequent, at most once or twice a year. During the study period, 36% of hospitals received at least one gynaecological surgical camp, and 33% received at least one orthopaedic surgical camp. District provision was nevertheless enhanced by these camps and until stable capacity is present, these visiting teams and camps remain important for some specific surgeries, though not Bellwether operations.

#### Orthopaedics

For operative orthopaedics, there was no documentation of treatment of open long bone fracture (Bellwether indicator). Simple closed fracture reduction and wound washouts were widely performed across many hospitals and are the most common operations performed; this begins to meet a basic trauma burden in remote districts.

Open fractures were not recorded as this was not a diagnostic term at use in Nepali hospitals. Wound debridement of open fractures may have been done and it is also likely that transfers of open fracture occurred without application of plaster stabilisation or wound washouts. However, without analysis of individual cases, it is not possible to know if a wound debridement is for a fracture or wound alone, nor what treatment was given before transfer. Transfer documentation was and remains poor.

Of note, external or internal fixation (major operative orthopaedics) took place only in hospital camps by visiting surgeons or at the two non-government district hospitals where orthopaedic surgeons were present. Orthopaedic operative volumes in those few hospitals were significant: open reduction or application of external fixator/traction were thus recorded as the sixth most common operation overall across all districts.

For all surgeries, in two hospitals, operations were not recorded throughout the whole year due to the absence of a surgeon. In several others, there were gaps of several months between operations throughout the year, either due to lack of cases or lack of a surgeon on staff.

### Human resources for surgery

**Fig 3:**
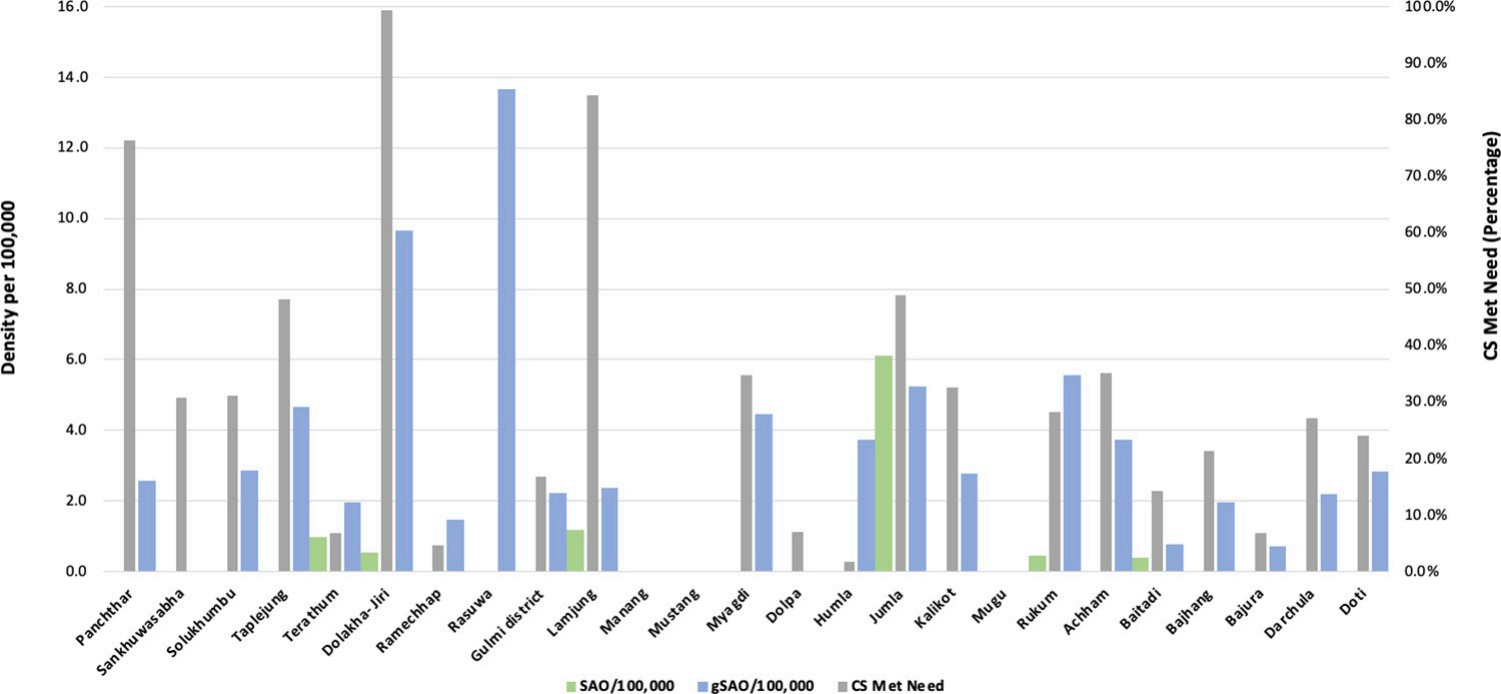
CS met need compared to gSAO and SAO densities per district.

The LCoGS target indicator for human resources for surgery has been defined as 20 specialist surgical, anaesthesiology, and obstetric doctors (SAO) /100,000 population [26], this being the level above which maternal mortality dramatically decreases. In this study, SAOs were very scarce. There were 13 SAOs over the entire study area; only 7 districts have SAO, of which 7 SAO are in one district with a medical college (KAHS) [Fig 3]. Specialist SAO density was thus very low at 0·4 per 100,000 for the whole study population.

The GoN has a policy to post generalist hospital doctors (MDGP) to provide essential surgery supported by non-doctor anaesthesia providers (anaesthesia assistants, AA) [32]. For CS, the MDGPs may be assisted or deputised by Medical Officers (MO) with advanced SBA level (ASBA) training; the latter doctors may even be the only surgical provider.

This study found that in these remote districts, the post-graduate doctor with surgical capability most usually present is an MDGP [Fig 3]. There were 22 MDGPs in 18 hospitals in 15 districts and assisted in anaesthesia by AAs, MOs, or another MDGP.

Using the metric of the generalist surgical team or generalist SAO (gSAO) across all study districts, a total of 108 gSAO are present and much more widely spread making gSAO density 8 times higher than SAO at 3·1 per 100,000.

At the time of the study in 2016, the LCOGS indicators were entirely SAO-specialist doctors, not generalist doctors and not paramedics. We coined the term gSAO to encompass other essential cadres actually delivering surgery at district hospitals–the key finding of our study and an innovative, effective solution to the lack of SAO staff across the study districts. We are pleased to see that the Utstein consensus on Global Surgery, obstetric and anaesthesia indicator definitions and reporting [33] re-defines LCOGS workforce indicators as the number of each of surgery, obstetric or anaesthesia providers who are actively practising, per 100000 population, and specifically includes non-specialist physician providers and non-physician practitioners of surgery, obstetric and anaesthesia care. For Nepal, these are our gSAO teams: MDGPs, ASBAs (MO with Advanced SBA training) and Anaesthesia Assistants.

#### CS met need

We analysed the Caesarean section met need compared to SAO and gSAO density by study district [Fig 3] and found a strong association between CS met need and gSAO density as opposed to SAO density. SAO density was very low in most districts suggesting that gSAOs were carrying out the majority of CS. The district of Rasuwa was an outlier where the gSAO density was high but CS was not being carried out.

### Surgical readiness or enabling environment for surgery

An adequate enabling environment for surgery, defined as having greater than 80% of all components on the data tool, was recorded in 46% of hospitals. 28% of hospitals had a functioning GA machine, although only 10% of hospitals could perform GA (both machine and halothane available). 74% of hospitals had a transfusion service consisting of either a blood bank or volunteer pool (“walking blood bank”), although half of all hospitals had no 24-hour blood service. X-ray was available 24 hours in the majority of hospitals (77%) [see S4 Fig]. The WHO Safe Surgery checklist was used in only 13% of study hospitals.

### Predictors of surgically functional hospitals

#### Predictors of a hospital’s ability to carry out Caesarean section

In order to better understand models of surgical functionality and success seen, hospitals were compared by numbers of Caesarean sections performed and any association with the type of facility, support received by the hospital, enabling environment of the hospital, and the presence of generalist surgical teams (gSAO) and specialist surgical teams (SAO) [Tables 1 and 2]. The presence of a generalist team, usually led by an MDGP, was significantly associated with performing a CS (Fisher’s Exact, p<0.001) whereas the type of support given to the hospital and the presence of specialist surgeons were not associated. Access to CS was also strongly associated with the presence of an adequate enabling environment at the hospital, defined as having at least 80% of the WHO Safe Surgery Checklist components like 24 hr transfusion service, and a functioning GA machine (Fisher’s exact, p<0.001). In the multivariate analysis, the presence of a generalist team (gSAO) was associated with a two-fold increase in access to Caesarean section (p = 0.032).

**Table 1:** Univariate associations predicting access to CS.

Predictor Variable	Access to CS		
**Type of Facility**	**Yes**	**No**	p-value*
District	23 (79.3%)	6 (20.7%)	0.043
Private	4 (40.0%)	6 (60.0%)	
**Support Received**			
Yes	23 (74.2%)	8 (25.8%)	0.221
No	4 (50.0%)	4 (50.0%)	
**Enabling Environment (>80%)**			
Yes	17 (94.4%)	1 (5.6%)	0.001
No	9 (42.9%)	12 (57.1%)	
**gSAO (n = 108)**			
Mean (SD)	3.88 (2.03)	0.54 (1.66)	<0.001
**SAO (n = 13)**			
Mean (SD)	0.46 (1.42)	0.07 (0.28)	0.342

*p-values yielded from 2-sided Fisher’s exact test (categorical variables) and 2-sample t-tests (continuous variables).

**Table 2:** Multivariate associations predicting access to CS.

Outcome Variable	Predictor Variable (Reference Category)	Adjusted Odds Ratio [95% CI]	p-value**
**CS Availability**	gSAO	2.06 [1.06, 3.99]	0.032
	SAO	1.59 [0.11, 23.06]	0.735
	Enabling Environment (80% or above)	6.05 [0.48, 75.87]	0.163

**p-values yielded from a multinomial logistic regression model.

### “Stepping stone” operations

“Stepping stone” operations are mid-level operations that require a more skilled and confident surgical team and may act as a “stepping stone” from CS to more complex, less common, and risky procedures (e.g. laparotomy) for a gSAO team. Procedures chosen for this analysis were inguinal hernia repair, total abdominal hysterectomy, and appendectomy. 13 hospitals doing stepping stone operations were also doing laparotomy but four hospitals doing stepping stone operations were not yet performing laparotomy [S5 Fig]. If a hospital did all 3 stepping stone surgeries, there was a significant association with the availability of laparotomy (p<0.001), [see S3 Table]. Sadly, stepping stone operations were few and infrequent. Nonetheless, these more complex operative skills represent a step-up for a district hospital surgical service on a pathway to higher capability.

### Perioperative mortality

Perioperative outcomes are an essential indicator of quality in surgical care [34–39], but were inconsistently recorded. Surgical mortality before hospital discharge (LCoGS target 4) was not recorded in any facility; 12 of the 39 hospitals recorded death in the OT itself. For all hospitals, this rate was 0·15% (2/1353) for CS and 2% (280/13954) for total operations. Clinical severity and late presentations may well contribute to this mortality but could not be determined; only 13% of hospitals used the WHO Safe Surgery Checklist.

The rate for newborn mortality recorded in the OT is 3% (45/1353); whether stillbirths or deaths after delivery cannot be disaggregated. Other outcomes or critical events were not recorded in the OT record.

### Access and referrals

A key LCoGS target is that 100% of the population are within 2 hours of a facility able to perform the Bellwether procedures [1]. For the study, Bellwether surgical access was calculated as the percentage of the overall total study population with the Bellwether procedures having been performed at least once in their district in the past year. Using this relatively crude and generous definition of access, for the total study population, 97% had access to CS, 22% had access to GI laparotomy, and 34·8% had access to major operative orthopaedics.

Access cannot be assumed to be reliably continuous 24h, 365 days a year but capability clearly exists and thus matches the new 2021 revised Utstein definition as the proportion of a country’s population with geographic access (within 2 hours) to a facility capable of providing surgical and anaesthesia care for the Bellwether procedures (Caesarean section, laparotomy, and surgical management of open long bone fracture)” [33]

However, operations were not recorded continuously throughout the year in several districts, 45% of districts referred for CS even though a CS had been performed in their district, and within-district travel would frequently be well over 2 hours and costly. Laparotomy and operative orthopaedic procedures in the districts were highly inconsistently performed with most orthopaedic procedures being carried out at camps organised outside the regular hospital services.

100% of the hospitals reported referral of surgical patients outside the district—45% of the hospitals reported having referred out CS cases due to unavailability of providers, 95% of the hospitals had referred out for GI laparotomy and 92% of the hospitals had referred out for open fractures cases. In 95% of hospitals, patients had to travel more than two hours to a surgically capable referral hospital, thus indicating a shortcoming in truly achieving the LCoGS target.

Data on transfers into and out of district hospitals are not well recorded. Across all hospitals, there was insufficient data about transfers; some patients are transferred without seeing any health worker, some are treated beforehand. This remains the case in many district hospitals (personal communication, Province 2).Open fractures and cases requiring emergency laparotomy were likely transferred. However, specific numbers are lacking.

### Needs/Expectations, barriers and solutions to more surgeries in rural and remote areas

We aimed to further elucidate and verify the quantitative findings from the survey through qualitative questions asked in a short interview format at the end of the survey. These questions asked the key respondents about the surgical service expectations and needs at the hospital, gaps or barriers faced in meeting those needs and possible solutions and recommendations. We have summarised the major themes that emerged from the qualitative questions in S4 Table and include some pertinent quotes below [S4 Table].

Caesarean section and orthopaedic surgeries were seen as significant areas of need, matching the Lancet Bellwether procedures:

“Caesarean section should be done here as Dolpa being a district inaccessible by road, a pregnant mother being referred to a higher centre is a big challenge due to unavailability of regular flights.” (Dolpa District Hospital)

One of the consistent barriers to surgery was the lack of human resources for surgery:

“Operating theatre setup is here but due to the lack of MDGP/ surgeon/ ObGyn all around the year, surgery cannot be performed.” (Dolpa District Hospital)

Comments on accessibility highlighted the multitude of factors that influence surgical access and patient choice. Some districts without good road infrastructure had issues with patients getting to the hospital for surgery while in other districts where road access was very good, patients preferred to bypass the district hospital and go to a higher centre:

“Because of easy availability of roads, patients want to go to a higher center.” (Panchthar District Hospital)

Lack of equipment and supplies was seen as another important barrier:

“Logistic supply, lack of Instruments, infrastructure, lack of general Anaesthesia…especially for GI surgery” (Myagdi District Hospital and others)

Community and medico-legal support were also mentioned by some of the interviewees, as violence against health care workers has become a worsening problem in Nepal:

“Lack of public faith” (Taplejung District Hospital)

“Patient and patient parties’ assurance; community awareness” (Rukum District hospital)

As for solutions, interviewees suggested removing the above-mentioned barriers to human resources, access, and equipment/logistics through increased training, retention packages for human resources, and increasing public awareness:

“Doctors working in remote areas should be given basic surgical training (despite their specialities) and laws should be flexible for these doctors in remote areas.” (Chaurjahari Hospital, Rukum)”

“Short-period orthopaedic training should be given to doctors and nursing staff.” (Bajhang District Hospital)

## Discussion

This is the first systematic, site-based study to map what surgical care looks like across Nepal’s remote rural districts. Whilst a long way below international targets, surgery, particularly essential CS, is happening in rural/remote Nepal. This is largely due to a unique programme of task-sharing by a family physician-led generalist surgical delivery team-the gSAO team. Such teams are available at a density eight times higher than specialists, are significantly associated with the delivery of CS, and are thus providing a pathway to effective surgical coverage.

The Lancet Commission on Global Surgery estimates that 5 billion people do not have access to safe, affordable surgical and anaesthesia care, particularly in LMICs, where nine of ten people cannot access basic surgical care [1]. Of the 313 million procedures undertaken worldwide each year, only 6% occur in the poorest countries [1]. Low operative volumes are associated with high case-fatality rates from common, treatable surgical conditions [2]. Unmet need is greatest in eastern, western, and central sub-Saharan Africa and South Asia [2, 3]. Based on the lack of specialist surgeons and anaesthesiologists and the remoteness of the districts and hospitals surveyed in this paper, we hypothesized that surgery, particularly major operations, like CS and laparotomy, would not be occurring in sufficient numbers in these districts.

Surprisingly, we found that surgery can be and is being performed in these remote districts. Overall rates of major operations compared with LCOGS targets are indeed low, but CS, the primary essential major operation at the district level, is being performed in many districts. Rates are better than the national mean in some districts, even meeting estimated needs in one district (Dolakha). Complex high-risk surgery such as laparotomy can be done; numbers are very low, but GI laparotomy has been performed by a generalist surgical team.

For the risky more complex operation of laparotomy, a risk across the surgical team including post-operative care, confidence may build from bravely doing one laparotomy close to an operation more familiar e.g. gynae laparotomy. This may well be true especially for the gSAO teams of MDGP and MO with ASBA training, and AA. GI laparotomy is perhaps more unpredictable but team confidence may grow from performing GI stepping stone procedures like appendectomy and inguinal hernia repair. They seem to mark a progression in surgical team confidence; this is particularly true of the gSAO teams and this pattern is still seen in Nepali district hospitals (personal observation, Province 1 and 2). It could be questioned whether doing only one laparotomy rarely is safe or maintains any peri-operative skill in such an emergency. However, this is an uncommon operation at district level, thus getting repetitive experience is difficult and more importantly, transfer incurs a big risk and cost to a patient and family.

Operative orthopaedics at government district hospitals was primarily delivered by visiting orthopaedic surgical camps. Generalist surgical teams led by MDGPs do manage simple fractures but usually do not do more complex operative orthopaedics. The recent 2021 Utsein review [33] recommended surgical management of open long bone fracture as an important indicator of emergency orthopaedic provision. Data points to record this key LCOGS indicator were not available in 2016 and remain systematically unrecorded across Nepal. In fact, is surgical management of open long bone fracture a useful metric of essential emergency orthopaedic provision across district hospitals in Nepal? Better indicators seem to be a progression from simple manipulation under anesthesia of closed fractures to wiring of fractures and internal nailing.

However, SAO density in these rural and remote districts is very low and it is clear that surgery in these districts is driven by generalist doctors (e.g. MDGP and MOs) and task-sharing health workers like anaesthesia assistants. Significantly, the presence of gSAOs (led by MDGP) at a health facility is positively associated with CS availability.

Given that our study was conducted in 2016, we decided to collect more recent data in 2022 to see if there were any major changes in SAO and gSAO density. In 2022, 23 of the original 39 hospitals were sent a short questionnaire and current district population densities were obtained. Although more specialists are being deployed with SAO density now at 1.0/100000 population, gSAO teams remain integral to surgical services with gSAO density at 2.4. In addition, despite the increase in numbers of SAOs, they are more concentrated in larger cities and hospitals as seen by the fact that 16 out of 27 SAOs were in a single hospital affiliated with a medical school. Please see S5 Table for a comparison for 2016 and 2022 data [S5 Table].

Successful models of healthcare task shifting and task sharing are widening globally. Non-specialist physicians (NSPs) providing essential surgical services at first-level hospitals have been described in other parts of the world [40]. A systematic review of NSPs revealed that surgical task-shifting/sharing to NSPs occurs across all country income groups; some provide surgical obstetrics, while others also provide a broader scope of surgical services [41]. Within LMIC countries, the majority are in sub-Saharan Africa. To ‘‘close the gap” in essential surgical services at the first-level hospital, more task-sharing needs to occur among both NSPs (like MDGPs) and non-physician clinicians (like AAs) [42–45]. Kim et al. argue that additional surgical training for family physicians, the key clinicians in primary care at rural or district hospitals, can play a critical role in reducing disparities in access to surgical, obstetric, and anaesthesia care in low- and middle-income countries and in rural or remote settings [44]. Our study is the first to describe the successful delivery of district surgical care, led by such family physicians.

The enabling environment for surgery was not adequate in many hospitals; however, Bellwether operations can be performed despite full provision. Interestingly, in our study, the presence of a generalist team (gSAO) was associated with a two-fold increase in access to Caesarean section (p = 0.032). However, the enabling environment for surgery at a hospital, defined as having at least 80% of the WHO Safe Surgery Checklist components like 24-hour transfusion service and functioning GA machine was only significant in the univariate analysis and not in the multivariate analysis, suggesting that human resources (gSAO) are the main drivers of surgical provision, and not equipment and supplies. These findings are very similar to what was seen in the Ugandan public sector where monthly operative volume was strongly predicted by the number of surgical, anaesthetic, and obstetric physician providers and not correlated with the availability of electricity, oxygen, light source, suction, blood, instruments, suture, gloves, intravenous fluid, or antibiotics [46].

While key LCoGS targets seem broadly applicable in Nepal, some local contexts must be taken into consideration. As anywhere, the 2-hour access target requires disaggregated population demographics [29, 47] and for Nepal, further clarification and consistency are required on the number of operations per 100,000 (definitions of major and minor, or even a surgical procedure, may differ from international classification) and case definitions of laparotomy and open fracture management need to be clearly defined. Minimum standards for assessing the enabling environment for surgery are an essential tool for assessing and building effective district surgery-international tools required adaptation for a Nepali context.

Finally, demonstrable effective coverage through gSAO teams means that in addition to SAO density, the gSAO density is an important metric to assess human resources for surgery: this is supported by the Utstein revised LCOGS definitions.

### Limitations

The quality of surgical provision and surgical outcomes were not addressed in the study and would be vital data to inform pathways to universal surgical provision. However, a lack of quality data is a problem in most LMICs, and interventions to improve data quality can be carried out with success as was done in an 8-week training and mentorship model programme in Ethiopia [45].

Additionally, the districts visited form a large sample of remote rural districts but did not include any Nepal plains (Terai) districts nor urban centers. A truer picture of access would additionally include measures of surgical availability over the whole year, an estimate of the burden for non-CS major surgery, patient financial expenditure, and geospatial population studies. Detailed data on referrals, patients bypassing district hospitals, and factors affecting patient decision-making would enrich the analysis.

### Policy implications

This study is the first to map successful pathways to essential surgical care in remote districts of Nepal and shows that successful district surgical teams can be composed of generalist teams led by a multi-skilled MDGP-the gSAO team.

Skilled human resources and the enabling environment for surgery must be urgently improved up to the minimum standard to provide full effective coverage across all districts.

Monitoring progression to effective surgical coverage must be embedded in HMIS and national reporting with prospective data collection based on adapted surgical data- tools, LCoGS international indicators, and locally applicable indicators such as gSAO teams. Until there are widespread specialist surgical SAO teams, gSAO teams should be supported to perform essential surgeries.

## Conclusion

Despite very low surgical specialist density and an often inadequate enabling environment, surgery can be done in Nepal’s remote districts. Generalist gSAO teams led by family physician MDGPs are providing essential surgery, with the essential district operation, CS, performed in significant numbers. gSAO density is eight times higher than specialists and they can undertake more complex operations than just CS alone. The family physician or MDGPs lead functional teams and are providing a pathway to effective surgical coverage in remote Nepal. This is vital evidence for the development of a national surgical plan for Nepal and other similar LMICs.

## Supporting information

S1 Table Examples of major and minor surgeries per Government of Nepal definitions. (PDF)

S2 Table Distance to nearest referral facility in another district. (PDF)

S3 Table Univariate associations between stepping stone procedure availability and laparotomy availability. (PDF)

S4 Table Summary of the qualitative responses. (PDF)

S5 Table SAO and gSAO density in 2016 vs 2022. (PDF)

S1 Fig Total major and minor surgeries performed per study district. (PDF)

S2 Fig Number of hospitals and study districts with capacity for essential surgeries. (PDF)

S3 Fig Distribution of operations as percent of total surgery performed, across all study districts. (PDF)

S4 Fig Presence of surgical readiness and enabling environment indicators across study hospitals. (PDF)

S5 Fig Hospitals performing stepping stone surgery, laparotomy, Caesarean sections. Each column represents a type of surgery done; x-axis secondary labels represent a hospital in that district.
(PDF)

S1 Data Dataset. (XLSX)
