# Supplementary material for: Pathways to effective surgical coverage in a lower-middle-income country: A multiple methods study of the family physician-led generalist surgical team in rural Nepal
Source: PLOS Glob Public Health. 2023 Feb 28;3(2):e0001510. doi: 10.1371/journal.pgph.0001510 (PMC10021892; doi:10.1371/journal.pgph.0001510)
Supplement: S1 Table — (PDF) [file pgph.0001510.s001.pdf]

S1 Table. Examples of major and minor surgeries per Government of Nepal definitions.

**Procedure**	**Categorization**	**Anaesthesia Provided**
Acute burn management - skin grafts	Major	
Amputation of limb	Major	Spinal, GA
Appendectomy	Major	
Burr holes	Major	
Caesarean section	Major	
Cholecystectomy	Major	
Cleft lip repair	Major	
Club foot repair	Major	
Colostomy	Major	
Hernia repair	Major	
Hysterectomy	Major	
Laparotomy	Major	
Open treatment of fracture (ORIF)	Major	
Repair of ano-rectal malformation	Major	
Repair of obstetric fistula	Major	
Shunt for hydrocephalus	Major	
Trauma-related amputation	Major	
Acute burn management	Minor	None, local
Acute burn management - debridement, fasciotomy	Minor	Ketamine
Biopsy	Minor	
Cataract extraction and insertion of intraocular lens	Minor	
Cervical cancer inspection	Minor	
Chest tube insertion	Minor	Any
Closed treatment of fracture (POP/MUA)	Minor	None, any
Dental abscess	Minor	None, local
Dilation and curettage	Minor	

Drainage of osteomyelitis or septic arthritis	Minor	
Escharotomy/fasciotomy	Minor	
External fixator/traction	Minor	
Eyelid surgery for trachoma	Minor	
Gastroscopy	Minor	
Gynaecologic suturing	Minor	Any
Hydrocoele	Minor	
Incision and drainage of abscess	Minor	Any
Male circumcision	Minor	
Relief of urinary obstruction	Minor	
Removal of foreign body (ear, eye, nose, throat)	Minor	Any
Surgical airway (cricothyroidotomy, tracheostomy)	Minor	
Tendon repair	Minor	Any
Treatment of open fracture (irrigation, debridement)	Minor	
Tubal ligation	Minor	
Urinary catherisation	Minor	
Vasectomy	Minor	
Wound suturing	Minor	Any
Acute burn management - skin grafts	Major	
