# Supplementary material for: Pathways to effective surgical coverage in a lower-middle-income country: A multiple methods study of the family physician-led generalist surgical team in rural Nepal
Source: PLOS Glob Public Health. 2023 Feb 28;3(2):e0001510. doi: 10.1371/journal.pgph.0001510 (PMC10021892; doi:10.1371/journal.pgph.0001510)
Supplement: S2 Table — (PDF) [file pgph.0001510.s002.pdf]

S2 Table. Distance to nearest referral facility in another district.

Hospital	District	Nearest referral facility in another district	Distance to referral facility (our assumption based on paper is that distance is >2 hours--that was our definition of remote district)	Time to travel
Panchthar District Hospital	Panchthar	BP Koirala Institute of Health Sciences	122 km	5 hr 11 min
District Hospital	Sankhuwasabha	BP Koirala Institute of Health Sciences	159 km	5 hr 18 min
Kunde Hospital	Solukhumbu	Kathmandu [Different Tertiary/Specialized hosptials]	272 km	8 hr 40 min
Lukla Hospital	Solukhumbu	Kathmandu [Different Tertiary/Specialized hosptials]	Flight	8 hr 40 min
Phaplu Hosptial	Solukhumbu	Kathmandu [Different Tertiary/Specialized hosptials]	272 km	8 hr 40 min
District Hospital	Taplejung	BP Koirala Institute of Health Sciences	194 km	7 hr 30 min
Terhathum District Hospital	Terathum	BP Koirala Institute of Health Sciences	492 km	13 hr 25 min
Community Hosptial	Dolakha	Patan (Patan Hospital)	179 km	6 hr 29 min
Gauri Shankar Hospital	Dolakha	Patan (Patan Hospital)	179 km	6 hr 29 min
Dolakha District Hospital, Jiri, Khadichaur	Dolakha	Patan (Patan Hospital)	180 km	5 hr 35 min
Primary Healthcare Center	Dolakha	Patan (Patan Hospital)	128 km	4 hr 4 min

Community Coperative Hosptial	Ramechhap	Patan (Patan Hospital)	140 km	4 hr 23 min
District hospital	Ramechhap	Patan (Patan Hospital)	139 km	4 hr 21 min
Manthali Primary Healthcare Center	Ramechhap	Patan (Patan Hospital)	127 km	3 hr 57 min
District Hospital	Rasuwa	Kathmandu [Different Tertiary/Specialized hosptials]	96.3 km	4 hr 10 min
Tamghas Hospital	Gulmi	Tansen Mission hospital (Palpa)	73.2 km	2 hr 35 min
Resunga Hospital	Gulmi	Tansen Mission hospital (Palpa)	77 km	3 hr 23 min
Community Hospital	Lamjung	Kathmandu [Different Tertiary/Specialized hosptials]	187 km	6 hr 8 min
District Hospital	Manang	Pokhara (Gandaki/Private Hospital)	168 km	7 hr 33 min
Mustang District hospital	Mustang	Pokhara (Gandaki/Private Hospital)	151 km	5 hr 50 min
District Hospital	Myagdi	Pokhara (Gandaki/Private Hospital)	85.7 km	2 hr 43 min
Private	Myagdi	Pokhara (Gandaki/Private Hospital)	85.7 km	2 hr 43 min
District Hospital	Dolpa	Nepalgunj (Bheri Hospital/Pvt Medical Collage)	Flight	No road access
District Hospital	Humla	Nepalgunj (Bheri Hospital/Pvt Medical Collage)	Flight	No road access

Karnali Academy of Health Sciences, Teaching Hospital	Jumla	Nepalgunj (Nepalgunj Medical College Teaching Hospital)	316 km	10 hr 17 min
Kalikot District Hospital	Kalikot	Dhangadi (Seti / Private Hospital)	387 km	11 hr 1 min
Mugu District Hospital	Mugu	Nepalgunj (Bheri Hospital/Pvt Medical Collage)	Flight	No road access
Rukum District Hospital	Rukum	Butwal (Lumbini Province Hospital)	299 km	8 hr 37 min
Churjhari hosptial	Rukum	Nepalgunj (Bheri Hospital/Pvt Medical Collage)	165 km	4 hr 38 km
District hospital	Achham	Nepalgunj (Bheri Hospital/Pvt Medical Collage)	257 km	7 hr 37 min
Bayanpata Hospital	Achham	Nepalgunj (Bheri Hospital/Pvt Medical Collage)	288 km	8 hr 47 min
District hospital	Baitadi	Nepalgunj (Bheri Hospital/Pvt Medical Collage)	362 km	9 hr 22 min
District hospital	Bajhang	Nepalgunj (Bheri Hospital/Pvt Medical Collage)	423 km	11 hr 28 min
Private	Bajhang	Nepalgunj (Bheri Hospital/Pvt Medical Collage)	423 km	11 hr 28 min
District hospital	Darchula	Nepalgunj (Bheri Hospital/Pvt Medical Collage)	482 km	13 hrs 33 min
District hospital	Darchula	Nepalgunj (Bheri Hospital/Pvt Medical Collage)	422 km	13 hrs 33 min

Primary Healthcare Center	Darchula	Nepalgunj (Bheri Hospital/Pvt Medical Collage)	391 km	10 hr 34 min
District hospital	Doti	Nepalgunj (Bheri Hospital/Pvt Medical Collage)	356 km	9 hr 17 min
Soojung Hospital Rajapur Doti	Doti	Nepalgunj (Bheri Hospital/Pvt Medical Collage)	356 km	9 hr 17 min
