# Supplementary material for: Pathways to effective surgical coverage in a lower-middle-income country: A multiple methods study of the family physician-led generalist surgical team in rural Nepal
Source: PLOS Glob Public Health. 2023 Feb 28;3(2):e0001510. doi: 10.1371/journal.pgph.0001510 (PMC10021892; doi:10.1371/journal.pgph.0001510)
Supplement: S3 Table — (PDF) [file pgph.0001510.s003.pdf]

S3 Table. Univariate Associations between Stepping Stone Procedure Availability and Laparotomy Availability.

	Laparotomy Available	Laparotomy Unavailable	p-value*
Stepping Stone Procedures Available	13 (92.9%)	1 (7.1%)	<0.001
Stepping Stone Procedures Unavailable	0 (0.0%)	25 (100.0%)	

*p-values yielded from Pearson's Chi-Square test of independence.
