# Supplementary material for: Pathways to effective surgical coverage in a lower-middle-income country: A multiple methods study of the family physician-led generalist surgical team in rural Nepal
Source: PLOS Glob Public Health. 2023 Feb 28;3(2):e0001510. doi: 10.1371/journal.pgph.0001510 (PMC10021892; doi:10.1371/journal.pgph.0001510)
Supplement: S4 Table — (PDF) [file pgph.0001510.s004.pdf]

S4 Table. Summary of the qualitative responses

Suggested surgical services (expectations and needs)	Gaps or barriers faced	Suggested solutions and recommendations
• Major procedures: CS, emCS, hollow viscus perforations, appendectomy, obstructed hernias, limb saving operation in case of trauma.<br>• Appendectomy, laparotomy (ectopic and bowel (SBO, perforation) (several hospitals), ruptured uterus.<br>• Hysterectomy (Vaginal, transabdominal), hydrocele, vasectomy, open cholecystectomy.<br>• Open chole, duodenal ulcer perforation, minor amputation (limb), Open reduction internal fixation (ORIF), closed reduction internal fixations (CRIF) [expectation, ambition going up?].<br>• IM nailing / external fixation<br>• Dental related surgical procedures	• Lack of adequate infrastructure- old building, discoloration of the walls, leaking roof, poor sanitation.<br>• Lack of adequate/proper supply<br>• Legal hurdles<br>• Lack of trained HR<br>• Geography, lack of access road and transportation, and climate<br>• Administrative issues & poor management<br>• Financial resources<br>• Supplies and logistics- such as blood bank<br>• Referral sites<br>• Lack of trust [increased public awareness]	• Stable power supplies<br>• Equipment and instrument<br>• Training for staff<br>• Financial compensation<br>• HR- NGO trained person<br>• CME<br>• Organise surgical camps locally/demand side and community support [trust]<br>• Supplies and other logistics.<br>• Expand services<br>• Retain highly skilled HR
There are a lot of emergency and lifesaving surgical needs in the rural areas of Nepal, in which deployment of surgical teams is highly useful. This includes major and minor general surgeries, ortho related and others, if supported by other skilled manpower, anaesthesia, and functional equipment.	These hospitals face a lot of systematic and logistical barriers including geography and weather conditions as Nepal is among the LMIC with difficult terrain.	There are always opportunities for improving the hospitals including the management of emergency and lifesaving surgical services, with the provision of gSOAs [MOs, MGDPs] in rural areas.
