# Supplementary material for: Pathways to effective surgical coverage in a lower-middle-income country: A multiple methods study of the family physician-led generalist surgical team in rural Nepal
Source: PLOS Glob Public Health. 2023 Feb 28;3(2):e0001510. doi: 10.1371/journal.pgph.0001510 (PMC10021892; doi:10.1371/journal.pgph.0001510)
Supplement: S5 Table — (PDF) [file pgph.0001510.s005.pdf]

S5 Table. SAO and gSAO density in 2016 vs 2022.

Summary table		
2016		
Team	Number	Per 100,000
SAO	13	0.4
gSAO	108	3.1
2022		
	Number	Per 100,000
SAO	27	1
gSAO	66	2.4
