# Supplementary material for: Pathways to effective surgical coverage in a lower-middle-income country: A multiple methods study of the family physician-led generalist surgical team in rural Nepal
Source: PLOS Glob Public Health. 2023 Feb 28;3(2):e0001510. doi: 10.1371/journal.pgph.0001510 (PMC10021892; doi:10.1371/journal.pgph.0001510)

S4 Fig. Presence of Surgical Readiness and Enabling Environment Indicators Across Study Hospitals.

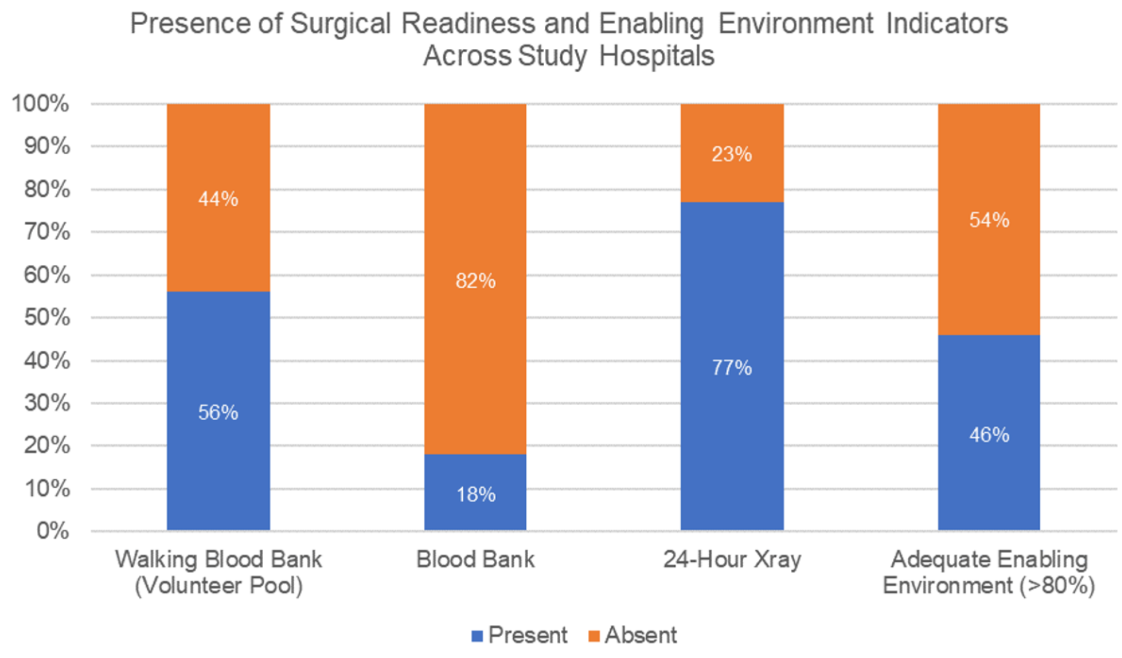

Supplement: S4 Fig — (PDF) [file pgph.0001510.s009.pdf]
