# Supplementary material for: Pathways to effective surgical coverage in a lower-middle-income country: A multiple methods study of the family physician-led generalist surgical team in rural Nepal
Source: PLOS Glob Public Health. 2023 Feb 28;3(2):e0001510. doi: 10.1371/journal.pgph.0001510 (PMC10021892; doi:10.1371/journal.pgph.0001510)

S5 Fig. Hospitals performing stepping stone surgery, Laparotomy, Caesarean sections. Each column represents a type of surgery done; x-axis secondary labels represent a hospital in that district.

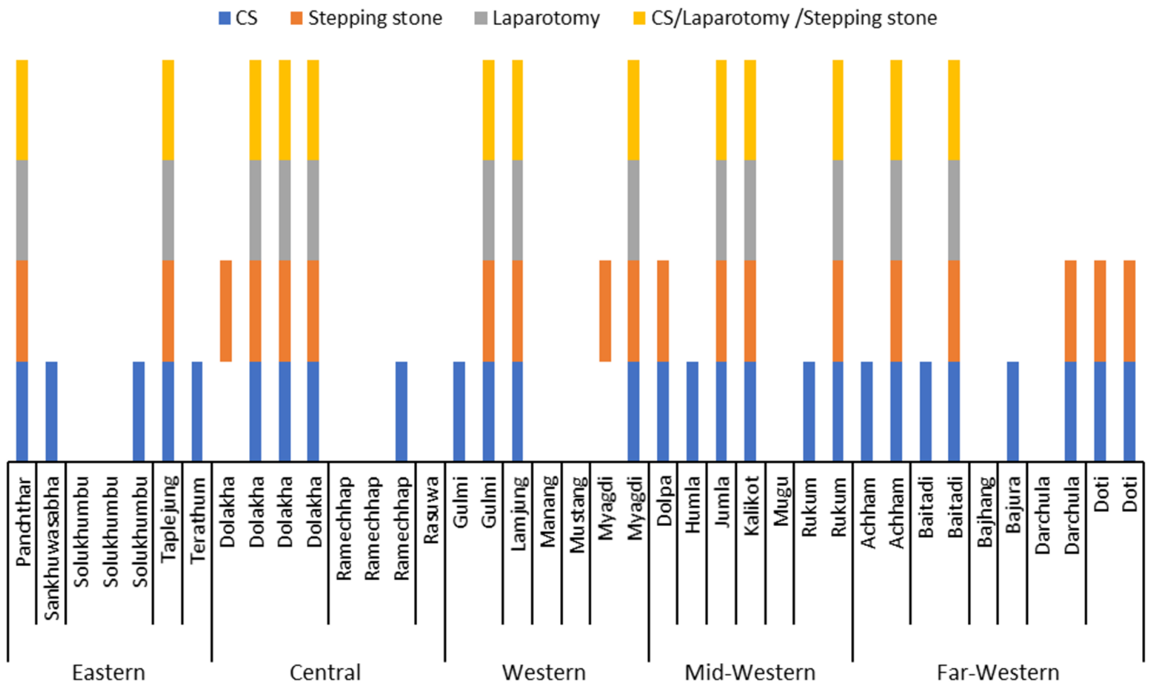

Supplement: S5 Fig — Each column represents a type of surgery done; x-axis secondary labels represent a hospital in that district. (PDF) [file pgph.0001510.s010.pdf]
